# Performance of an interferon-γ release assay-based test for cell-mediated immunity to SARS-CoV-2

**DOI:** 10.3389/fimmu.2023.1069968

**Published:** 2023-02-16

**Authors:** Luís Fonseca Brito, Silvia Tödter, Julian Kottlau, Kathrin Cermann, Anthea Spier, Elina Petersen, Ines Schäfer, Raphael Twerenbold, Martin Aepfelbacher, Marc Lütgehetmann, Felix R. Stahl

**Affiliations:** ^1^ Institute of Clinical Chemistry and Laboratory Medicine, University Medical Center Hamburg-Eppendorf, Hamburg, Germany; ^2^ Department of Virus-Host-Interaction, Leibniz Institute of Virology, Hamburg, Germany; ^3^ Institute of Microbiology, University Medical Center Hamburg-Eppendorf, Hamburg, Germany; ^4^ Department of Cardiology, University Heart and Vascular Center, University Medical Center Hamburg-Eppendorf, Hamburg, Germany; ^5^ Population Health Research Department, University Heart and Vascular Center, Hamburg, Germany; ^6^ German Center for Cardiovascular Research (DZHK) Partner Site Hamburg–Kiel–Lübeck, Hamburg, Germany

**Keywords:** coronavirus, COVID-19, interferon, interferon gamma release assay, QuantiFERON, T cell, SARS-CoV-2

## Abstract

In search for immunological correlates of protection against acute coronavirus disease 2019 (COVID-19) there is a need for high through-put assays for cell-mediated immunity (CMI) to severe acute respiratory syndrome coronavirus 2 (SARS-CoV-2) infection. We established an interferon-γ release assay -based test for detection of CMI against SARS-CoV-2 spike (S) or nucleocapsid (NC) peptides. Blood samples obtained from 549 healthy or convalescent individuals were measured for interferon-γ (IFN-γ) production after peptide stimulation using a certified chemiluminescence immunoassay. Test performance was calculated applying cutoff values with the highest Youden indices in receiver-operating-characteristics curve analysis and compared to a commercially available serologic test. Potential confounders and clinical correlates were assessed for all test systems. 522 samples obtained from 378 convalescent in median 298 days after PCR-confirmed SARS-CoV-2 infection and 144 healthy control individuals were included in the final analysis. CMI testing had a sensitivity and specificity of up to 89% and 74% for S peptides and 89% and 91% for NC peptides, respectively. High white blood cell counts correlated negatively with IFN-γ responses but there was no CMI decay in samples obtained up to one year after recovery. Severe clinical symptoms at time of acute infection were associated with higher measures of adaptive immunity and reported hair loss at time of examination. This laboratory-developed test for CMI to SARS-CoV-2 NC peptides exhibits excellent test performance, is suitable for high through-put routine diagnostics, and should be evaluated for clinical outcome prediction in prospective pathogen re-exposure.

## Introduction

Severe acute respiratory syndrome coronavirus 2 (SARS-CoV-2) infection induces a strong adaptive immune response including production of virus-specific antibodies and T cell immunity (1). Accordingly, besides direct detection of acute SARS-CoV-2 infection *via* polymerase chain reaction (PCR) or pathogen antigens *via* rapid lateral flow tests, indirect measures such as detection of endogenous antibodies to SARS-CoV-2 are useful to assess prior pathogen exposure or vaccination (2, 3). Detection of antigen-specific antibodies to epitopes of the SARS-CoV-2 spike (S) or nucleocapsid (NC) proteins are an established diagnostic approach to provide evidence of immunity after infection or vaccination and there are numerous commercially manufactured diagnostic tests available. In fact, effectiveness of antibodies to neutralize virus replication *in vitro* have been used to assess vaccine responses and predict protection from coronavirus disease 2019 (COVID-19) (4–7). Although the presence of anti-SARS-CoV-2 antibodies correlates with protection from severe COVID-19, there is strong evidence that antibodies do not mediate sterile immunity and prevent from infection or re-infection with this pathogen (8). The underlying mechanism of protection is likely mediated by additional components of the immune system and in this respect, T cell immunity has been implied as a critical determinant to protect from COVID-19-related hospitalization and death (9–11). High-throughput cellular assays to measure adaptive SARS-CoV-2 immunity are not commonly requested by physicians and in general less available than serologic testing. Potential reasons are higher costs, extended sample processing times, a more delicate pre-analytic process, and the necessity for higher efforts in developing cell-mediated immunity (CMI) assays. Despite these disadvantages there is a general need to assess cell-mediated adaptive immunity to specific pathogens such as *Mycobacterium tuberculosis* (12), for individuals who cannot acquire a sufficient humoral immune response due to underlying conditions, or to evaluate responses to chronic infectious diseases (13, 14). Several aspects such as potential correlates of protection from re-infection, broad range recognition of antigens, and detection of long-term immunity argue that SARS-CoV-2 CMI-based tests may be critical for upcoming pandemic-related clinical problems (15).

Here, we used an interferon-γ release assay (IGRA) that uses peptide pools of the S and NC proteins together with a certified *in vitro* diagnostic analyzer to detect SARS-CoV-2 CMI in a large clinical cohort. We assessed CMI assay performance and potential confounders, compared these results with a commercially available test for anti-NC antibodies, and correlated these measures of adaptive immunity with clinical symptoms.

## Materials and methods

### Clinical cohort

This study was performed similarly to the post-SARS-CoV-2 cohort previously reported in (16). Individuals living in the metropolitan area of Hamburg, Germany, who had a confirmed positive polymerase chain reaction (PCR) test for SARS-CoV-2 at least 2 months prior to study enrollment were included as convalescent individuals. In parallel, participants of the Hamburg City Health Study (HCHS) (17) with no history of previous SARS-Cov-2 infection and were enrolled during the same time period served as healthy controls. Participants without history of PCR-confirmed SARS-CoV-2 infection but a positive antibody test to SARS-CoV-2 nucleocapsid protein were not excluded from the healthy control group. Participants were 43 - 80 years old at the time of recruitment. All individuals, underwent the identical clinical examination program of the HCHS, which included a blood withdrawal and a questionnaire (only convalescent group) between November 16^th^ 2020 and April 28^th^ 2021. Categorization of disease severity at time of acute infection and potential long COVID-19 symptoms was based on information provided by participants in this questionnaire. Potential re-exposition after a primary SARS-CoV-2 infection was not addressed in the convalescent group. At the time of study enrolment, all participants provided written informed consent. The local ethics committee (State of Hamburg Chamber of Medical Practitioners, PV5131) had no objections against recruitment of post-SARS-CoV-2 individuals as an extension of the HCHS, and the study was conducted in compliance with the Declaration of Helsinki.

### CMI

Peptide pools of overlapping 15-mer sequences covering the complete SARS-CoV-2 nucleocapsid (PepTivator^®^ SARS-CoV-2 Prot_N, Miltenyi Biotec), immunodominant sequence domains of SARS-CoV-2 spike (PepTivator^®^ SARS-CoV-2 Prot_S, Miltenyi Biotec) protein, HCMV IE1 (PepTivator^®^ CMV IE-1, Miltenyi Biotec) and pp65 (PepTivator^®^ CMV pp65, Miltenyi Biotec) proteins were used for stimulation of peripheral blood T cells. Within the first 24h after blood collection, 800µL heparinized whole blood was mixed with either phosphate buffered saline (negative control), or 50 ng/mL phorbol-12-myristate-13-acetate (PMA) together with 500 ng/mL ionomycin (positive control), or 1.25 µg/mL SARS-CoV-2 peptides, or 0.625 µg/mL HCMV IE1 together with 0.625 µg/mL HCMV pp65 peptides for 24h at 37°C. IFN-γ concentration was measured in supernatants with a chemiluminescence immunoassay (QuantiFERON^®^) on a LIAISON^®^ XL analyzer (DiaSorin), as recommended by the manufacturer.

### Antibodies, clinical chemistry, hematology

Clinical chemistry and hematology parameters were determined with Atellica^®^ Solution and ADVIA 2120i (Siemens Healthineers), respectively. Anti-NC antibodies (Elecsys anti-SARS-CoV-2) were measured with cobas^®^ e411 (Roche) (18), as recommended by the manufacturer. The majority of recruited convalescent individuals was tested at the University Medical Center Hamburg-Eppendorf for acute SARS-CoV-2 infection with PCR as described in (19) and only these were included for correlation of Ct values to adaptive immunity.

### Statistical analysis

Statistical analysis was performed with Prism (GraphPad) using the tests as indicated. Dimensionality reduction was performed with R (version 4.2.1) using the Rtsne package (version 0.16).

## Results

**Figure 1:**
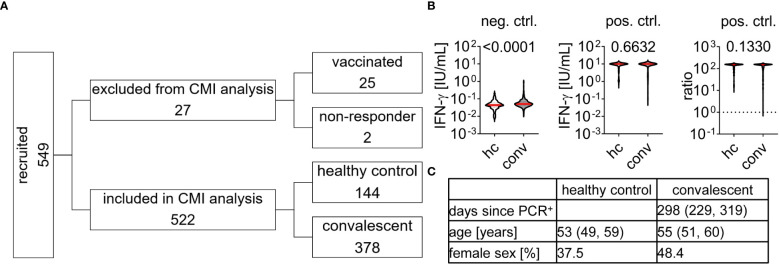
Study cohort characteristics and Interferon-ɣ release upon stimulation with control agents **(A)** Individuals were recruited and tested for cell-mediated immunity (CMI) to SARS-CoV-2 as indicated. 27 samples were excluded, 25 due to prior vaccination and two because they did not pass positive control requirements. 522 samples were included in CMI test performance analysis with cohort characteristics as described in **(C)**, data is provided by median and interquartile range. **(B)** Interferon (IFN)-ɣ concentration of all 549 samples in response to negative (neg. ctrl., buffer) or positive control (pos. ctrl., mitogen) stimulation. Dotted line in pos. ctrl. ratio indicates cut-off of 1 which need to be passed for inclusion in final CMI analysis. Differences between healthy control (hc) and convalescent (conv) group were assessed by Mann Whitney test and p-value is provided above each diagram.

We acquired CMI to SARS-CoV-2 S and NC peptides from 549 individuals recruited 62 to 386 days post infection from November 16^th^ 2020 to April 28^th^ 2021 and analyzed IFN-ɣ concentration in blood samples stimulated with either a negative (buffer) or positive (mitogen stimulation) control (**Figures 1A, B**). We found a higher median baseline IFN-ɣ concentration in negative control samples of convalescent individuals than in the healthy control group (0.0509 and 0.0437 IU/ml, p < 0.0001) whereas responses to mitogen stimulation was generally strong and at the upper measurement range (**Figure 1B**). In order to identify individual samples that respond insufficiently to mitogen stimulation we calculated the ratio between positive and negative control values and excluded samples with a value below 1.0 as “non-responder”. 522 samples obtained from 378 convalescent patients and 144 controls were included in the final analysis for test quality characteristics of the laboratory developed CMI tests (**Figure 1A**). In convalescent individuals, blood was collected in median 298 [Q_1_ 229, Q_3_ 319] days after the first PCR-confirmed SARS-CoV-2 infection (**Figure 1C**). The median age was 55 [Q_1_ 51, Q_3_ 60] years in convalescents and 53 [Q_1_ 49, Q3 59] years in controls. The healthy control group contained less female individuals than the convalescent group (37.5% *vs*. 48.4%). Sex had no effect on baseline IFN-ɣ concentration, positive controls, and subsequent CMI testing as described below (data not shown and **Table 1**). 27 samples were excluded from further analysis, 25 due to prior vaccination, and two samples did not pass positive control criteria (**Figure 1A**).

**Table 1:** Summary of potential confounders for anti-SARS-CoV-2 CMI and antibody testing (A) Correlation or direct comparison of cohort characteristics and additional laboratory parameters to measurements of SARS-CoV-2 CMI and antibody testing.

parameter		n	CMI S IU		CMI S ratio		CMI NC IU		CMI NC ratio			anti-NC	
			Spearman r	*p*	Spearman r	*p*	Spearman r	*p*	Spearman r	*p*	n	Spearman r	*p*
**days since PCR^+^**		365	-0.03444	0.5119	-0.02643	0.6147	-0.05616	0.2845	-0.0488	0.3525	347	-0.1987	***0.0002***
**age (years)**		378	0.1162	***0.0239***	0.1114	***0.0303***	0.06803	0.1869	0.06021	0.2429	360	0.2914	***<0.0001***
**BMI (kg/m^2^)**		352	0.01789	0.7381	0.02701	0.6135	0.01633	0.7601	0.02976	0.5779	336	0.2199	***<0.0001***
**WBC (counts/µl)**		370	-0.251	***<0.0001***	-0.2532	***<0.0001***	-0.3027	***<0.0001***	-0.3059	***<0.0001***	352	-0.01431	0.789
**PCR Ct value**		178	0.09298	0.2171	0.08094	0.2828	0.1238	0.0996	0.1044	0.1655	171	0.04558	0.5538
**Creatinine (mg/dl)**		368	-0.04805	0.358	-0.05293	0.3112	-0.01182	0.8212	-0.01016	0.8461	351	0.04649	0.3852
**cystatin C (mg/l)**		352	-0.0734	0.1694	-0.1075	0.0439	-0.07122	0.1825	-0.09972	0.0616	339	0.1236	***0.0229***
			median (IQR)	*p*	median (IQR)	*p*	median (IQR)	*p*	median (IQR)	*p*		median (IQR)	*p*
**sex**	female	183	0.2410 (0.124., 0.7870)	0.6502	3.554 (1.692, 10.49)	0.6349	0.6640 (0.1930, 1.500)	0.6246	8.406 (2.504, 20.62)	0.6655	170	30.18 (7.668, 81.619)	0.7898
	male	195	0.2960 (0.1260, 0.7280)		3.831 (1.754, 9.554)		0.6510 (0.2320, 1.700)		7.738 (3.462, 21.81)		186	26.83 (9.295, 81.34)	
**diabetes**	yes	24	0.2590 (0.09698, 0.6925)	0.6320	3.854 (1.492, 7.391)	0.7326	0.4555 (0.2430, 1.593)	0.7803	6.419 (3.738, 17.34)	0.8857	21	50.86 (19.69, 146.8)	***0.0254***
	no	336	0.2615 (0.1250, 0.7650)		3.697 (1.742, 10.45)		0.6795 (0.2083, 1.670)		8.710 (2.808, 21.52)		322	26.55 (7.913, 80.00)	
**dyslipidemia**	yes	77	0.2480 (0.1245, 0.7865)	0.9261	3.600 (1.777, 9.410)	0.6651	0.5030 (0.2040, 1.600)	0.4917	7.738 (2.857, 20.81)	0.7756	73	45.12 (16.49, 99.57)	***0.0230***
	no	285	0.2620 (0.1245, 0.7480)		3.815 (1.715, 10.41)		0.7030 (0.2100, 1.670)		8.631 (2.961, 21.31)		273	24.13 (7.320, 77.55)	
**smoker**	yes	32	0.2485 (1.563, 0.5490)	0.9848	3.531 (2.359, 6.802)	0.6610	0.5235 (0.2778, 1.270)	0.6997	7.132 (2.950, 16.38)	0.4194	31	26.98 (13.63, 69.17)	0.9355
	no	343	0.2610 (0.1240, 0.7730)		3.815 (1.692, 11.04)		0.6810 (0.2120, 1.670)		8.843 (3.000, 21.81)		327	28.10 (7.800, 81.89)	
**HCMV**	positive	17	0.4220 (0.1895, 0.8840)	0.7471	4.015 (2.675, 13.60)	0.9614	0.7030 (0.2820, 2.000)	0.4046	8.843 (4.100, 28.38)	0.5507	17	66.71 (26.01, 83.03)	0.0705
	negative	14	0.3240 (0.1748, 1.230)		4.985 (2.688, 18.92)		0.4170 (0.2278, 1.595)		6.415 (3.504, 24.54)		10	28.99 (5.998, 54.80)	

Number of samples (n) included for analysis is provided for each parameter. Statistical parameters are provided as indicated, for comparative analysis of two groups were assessed by Mann Whitney test. BMI, body mass index; (Ct) cycle threshold; HCMV, human cytomegalovirus; (PCR), polymerase chain reaction; (WBC) white blood counts. Days since PCR+ = days since first positive SARS-CoV-2 detection via PCR. Measures with statistical differences are highlighted in bold and italic.

**Figure 2:**
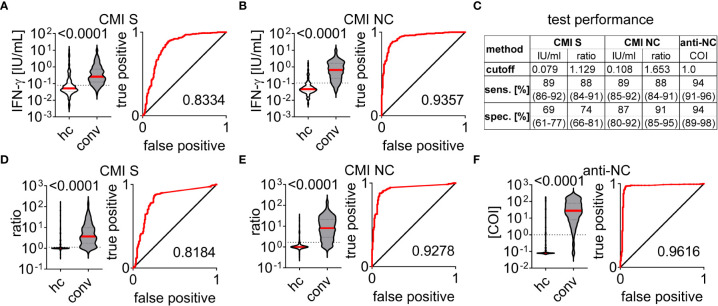
Test performance characteristics of cell-mediated immunity and antibody testing to SARS-CoV-2 Cell-mediated immunity (CMI) measured in healthy control (hc) and convalescent (conv) groups by supernatant interferon (IFN)-ɣ concentration in response to stimulation with peptide pools covering sequences of the SARS-CoV-2 **(A)** spike (S) or **(B)** nucleocapsid (NC) proteins. Ratios to respective negative controls are depicted in **(D)** for S- and **(E)** for NC- stimulated samples. **(C)** Test performance characteristics for CMI and antibody tests determined for cutoff values with highest Youden indices or provided by commercial manufacturer (anti-NC). Sensitivity (sens.) and specificity (spec.) are provided in median and interquartile range for each test application. **(F)** Anti-NC antibody titers provided as cutoff indices (COI). A+B, **(D–F)** Differences between healthy control (hc) and convalescent (conv) group were assessed by the Mann-Whitney test and p-value is provided above each diagram, dotted lines indicate corresponding cutoff value. Receiver operating characteristic curves and according area under the curve are provided for each test system.

IFN-ɣ concentration in blood samples stimulated with either SARS-CoV-2 spike (S) or nucleocapsid (NC) peptide pools were significantly higher in the convalescent than in the healthy control group (**Figures 2A, B**). We applied Receiver-Operating-Characteristics (ROC) curves to test for diagnostic abilities and found area under curve values of 0.8334 (0.7889 - 0.8779, 95%CI) and 0.9357 (0.9125 - 0.9589, 95%CI) for S and NC stimulated samples, respectively (**Figures 2A, B**). Accordingly, using cutoff values with the highest Youden indices in ROC analysis we found a sensitivity and specificity of 89% (86 - 92%, 95%CI) and 69% (61 - 77%, 95%CI) for the CMI test using S peptides whereas stimulation with NC peptides had the same sensitivity of 89% (85 - 92%, 95%CI) but a superior specificity of 87% (80 - 92%, 95%CI) (**Figure 2C**). We incorporated the differences in baseline IFN-ɣ concentration by calculating ratios of values measured in stimulated to respective negative control samples and re-analyzed the dataset (**Figures 2D, E**). Using this approach exhibited area under curve values of 0.8184 (0.7736 - 0.8633, 95%CI) and 0.9278 (0.9022 - 0.9534, 95%CI) for S and NC CMI testing (**Figures 2D, E**). We found a decrease of sensitivity to 88% for both, S (84 - 91%, 95%CI) and NC (84 - 91%, 95%CI) CMI testing, whereas specificity increased to 74% (66 - 81%, 95%CI) and 91% (85 - 95%, 95%CI), respectively (**Figure 2C**). Within this cohort we additionally performed anti-NC antibody testing of 364 convalescent patients and 140 healthy controls and calculated test performance characteristics according to the manufacturer’s cutoff index (COI) (**Figure 2F**). We found an area under curve value of 0.9616 (0.9375 to 0.9857, 95%CI) with a sensitivity and specificity of 94% (91 - 96%, 95%CI) and 94% (89 - 98%, 95%CI), respectively (**Figures 2C, F**). Together, detection of CMI against S and NC peptides exhibited very good to excellent diagnostic performance for detection of previous exposure to SARS-CoV-2. CMI testing with peptides of the NC was superior to those of the S protein and incorporation of baseline IFN-ɣ concentration improved diagnostic specificity of both tests. Nevertheless, all test systems analyzed here apparently exhibited false positive and false negative results.

**Figure 3:**
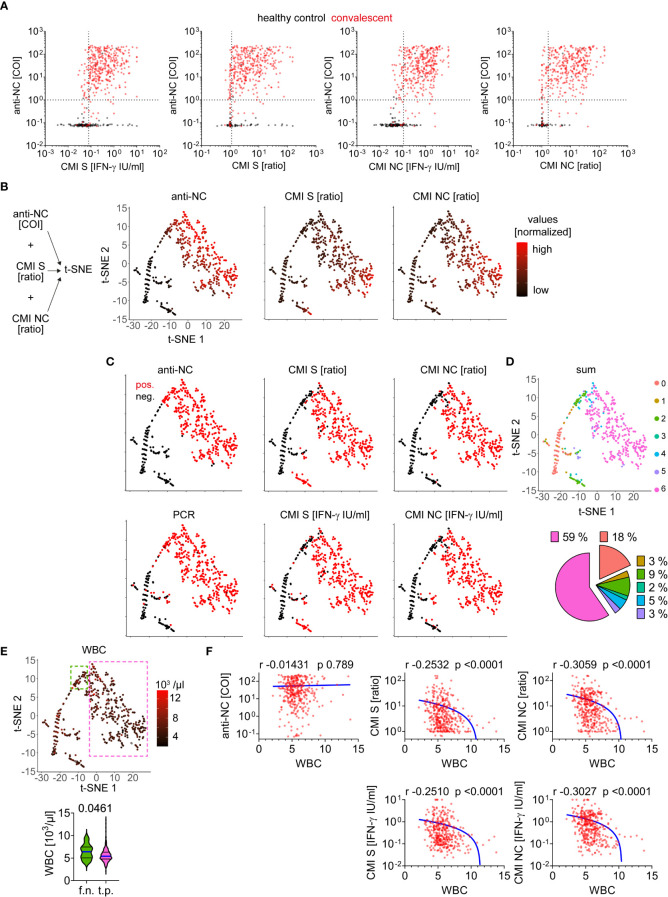
Comparative analysis of test applications for detection of SARS-CoV-2 infection **(A)** Absolute values measured for each CMI test and anti-NC are depicted as indicated. Dotted lines indicate cutoff values as calculated or provided by commercial manufacturer (anti-NC). **(B)** Data integration approach for dimensionality reduction and illustration of values (normalized) for each measured sample as indicated. **(C)** Test result as positive (pos.) or negative (neg.) after evaluation using calculated or provided cutoffs for each test system as indicated. **(D)** Concordance between test results of anti-NC, CMI S and NC ratios as well as absolute IFN-ɣ values, and PCR by summation of each result (negative = 0 and positive = 1) and superimposition in t-SNE plot as calculated in B). The pie chart illustrates the relative distribution of 100% agreement between test results (sum of 0 = 18% or 6 = 59%) as well as discrepancies (1 to 5). **(E)** Absolute number of white blood cell (WBC) counts measured in individuals at the same time as acquisition of anti-SARS-CoV-2 adaptive immunity testing. Values are superimposed in t-SNE plot and samples with high test concordance (sum of 6) considered as true positive (t.p) are framed in pink whereas samples with discordance between CMI tests versus antibody and PCR test (a fraction of the group with a sum of 2) are considered as false negative (f.n.) framed in green. Absolute WBC counts for these two subgroups are provided below, differences were assessed by Mann Whitney test and p-value is provided. **(F)** Correlation of WBC counts versus measurements as indicated. Calculated Spearman correlation results are provided for each diagram, lines indicate simple non-linear regression curves.

We aimed to analyze correlative features of the various techniques for detection of SARS-CoV-2 exposure. We found several samples of the healthy control group to be false positive in CMI measurements but true negative by anti-NC testing and vice versa (**Figure 3A**). Some samples obtained from convalescent patients could be detected as positive by anti-NC only but not by CMI testing and a few were detected by CMI testing only but were negative for anti-NC antibodies. Interestingly, several samples of the healthy control group tested positive for both CMI and antibodies. To gain more insight we used a dimensional reduction approach for visualizing the values of anti-NC, CMI S and NC ratios and found the samples to position in two main and several minor clusters (**Figure 3B**). Next, we superimposed the test results of all diagnostic methods in a binary format (positive or negative) using the previously calculated cutoff values (**Figure 3C**). Summation of the test results (negative = 0 and positive = 1) allowed visualizing concordance between the diagnostic tests (**Figure 3D**). We found 77% of the samples to have a sum of 6 or 0, implying a definite classification of samples to be obtained by patients either with or without prior SARS-CoV-2 infection, respectively. This approach allowed detection of several samples that were categorized as positive only by the CMI S test explaining the lower test specificity if compared to CMI NC (**Figures 3C, D**). Moreover, numerous samples obtained from convalescent patients were measured as positive *via* anti-NC, albeit with a rather low COI, but were below the CMI S and NC cutoff values (**Figures 3B–D**). Hypothesizing a confounding factor to be responsible for this observation, we analyzed additional routine laboratory diagnostic parameters for correlation to anti-NC or CMI measurements. We found higher white blood cell (WBC) counts in these false negative than in true positive CMI tests (**Figure 3E**). In general, there was a negative correlation between WBC counts and CMI IFN-ɣ responses (**Figure 3F**; **Table 1**). Accordingly, high WBC counts seemed to be a disruptive factor for CMI testing whereas anti-NC titers were not affected.

**Figure 4:**
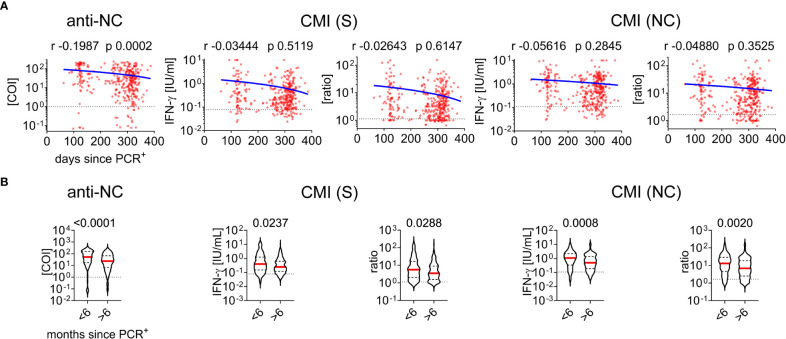
Cell-mediated immunity and antibody testing to SARS-CoV-2 in dependence of time between acute infection and measurement **(A)** Correlation of days since positive PCR testing versus measurements of CMI and antibodies as indicated. Calculated Spearman correlation results are provided for each diagram, blue lines indicate simple non-linear regression curves, dotted lines indicate cut-off values. **(B)** Comparative analysis of immunity in individuals infected less or more than six months before data acquisition. The Mann-Whitney test and p-value is provided above each diagram, dotted lines indicate corresponding cutoff value.

Next, we further analyzed cohort characteristics and various diagnostic laboratory parameters of the convalescent group for effects on SARS-CoV-2 antibody or CMI testing (**Table 1**). There was a negative correlation between anti-NC titers and the time since positive PCR testing for SARS-CoV-2 whereas CMI testing was not significantly affected (**Figure 4A**; **Table 1**). Next, we split the cohort into early and late time post infection to analyze for potential differences in immunity. We found significantly higher antibody and IFN-ɣ responses in individuals who were infected less the six months before blood analysis than in those who had recovered more than 6 moths (**Figure 4B**). In contrast, there was no correlation between PCR cycle threshold (Ct) values and measures of adaptive immunity indicating that virus loads at time of acute infection had no effect on the test systems applied in this study (**Table 1**). Moreover, we found sex, body mass index (BMI), diabetes, dyslipidemia, or smoking to have no robust effect on CMI testing although there was a minor significant positive correlation between CMI S testing and age (**Table 1**). In contrast, there was a positive correlation between age, BMI, and cystatin C with anti-NC titers. Moreover, samples obtained from patients with diabetes or dyslipidemia exhibited higher anti-NC titers indicating that metabolic aberrations were associated with higher antibody responses. We additionally screened 31 samples for cytomegalovirus (HCMV) seroprevalence and excluded any effect on the diagnostic interpretation of CMI testing. However, we found a significantly higher median baseline IFN-γ concentration in negative control samples of HCMV^+^ than HCMV^-^ individuals (0.06460 versus 0.04630 [IFN-ɣ IU/ml], p = *0.0268*) and a trend to higher median anti-NC antibody titers in HCMV^+^ individuals (66.71 versus 28.99 [COI], p = 0.0705) (**Table 1**). Together, humoral immunity to SARS-CoV-2 appeared to be affected by several conditions that had no significant impact on testing for CMI.

**Figure 5:**
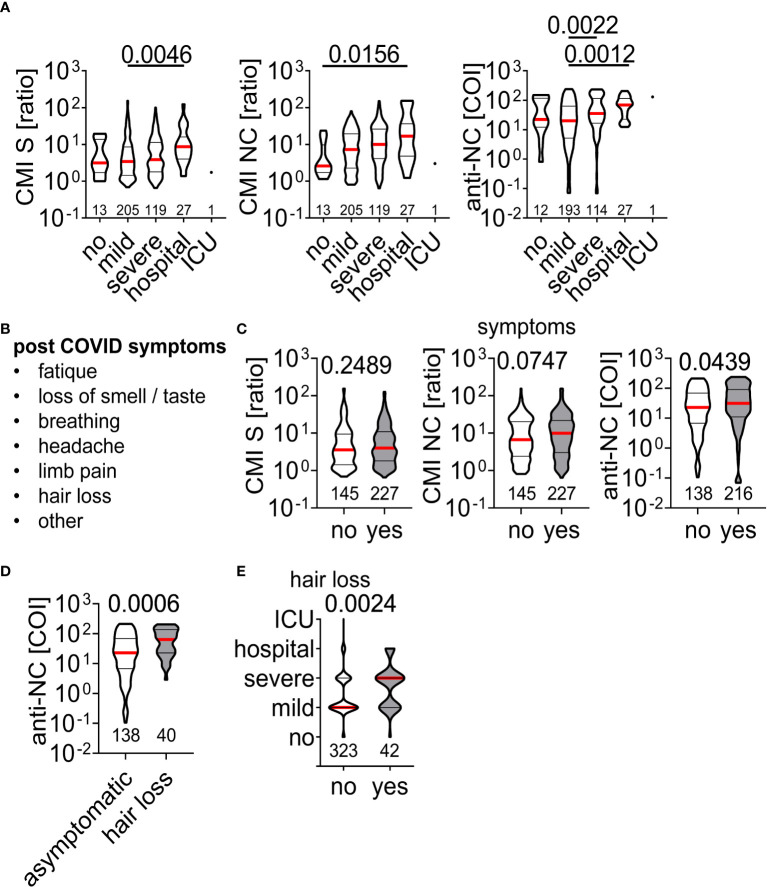
Association of acute and chronic clinical course of SARS-CoV-2 infection on measurements of adaptive immunity **(A)** Clinical course during acute SARS-CoV-2 infection with corresponding IFN-ɣ responses to peptide stimulation or antibody titers. Difference between groups was assessed by Kruskal-Wallis with Dunn’s multiple comparisons test and p-values below 0.05 are provided above each diagram. The number of individuals per group included in the analysis are provided within the diagrams. **(B)** List of post COVID symptoms retrieved in the questionnaire at time of sample acquisition and **(C)** test results in dependence of absence (no) or presence (yes) of symptoms. **(D)** Anti-NC titers of asymptomatic individuals and those who developed hair loss post SARS-CoV-2 infection. **(E)** Clinical course during acute SARS-CoV-2 infection of individuals with absence (no) or presence (yes) of hair loss. Differences in **(C-E)** were assessed by Mann Whitney test and p-value is provided.

Finally, we aimed to correlate clinical symptoms present at time of acute infection or blood withdrawal with IFN-ɣ production and antibody titers. There were higher responses in CMI testing with S and NC peptides as well as anti-NC titers for patients who had severe symptoms or were admitted to hospital during the acute infection phase (**Figure 5A**). Convalescent individuals were asked for potential long COVID-19 symptoms (**Figure 5B**) at time of visit for CMI testing but there was no difference in IFN-ɣ production in CMI testing between asymptomatic or symptomatic individuals (**Figure 5C**). In contrast, we found higher anti-NC titers in symptomatic than asymptomatic patients (**Figure 5C**), due to higher anti-NC titers in those who developed hair loss several weeks after acute infection (**Figure 5D**). Moreover, patients who exhibited severe symptoms at time of acute SARS-CoV-2 infection had an increased risk of developing hair loss (**Figure 5E**). Other post COVID-19 symptoms were not associated with the applied measures of adaptive immunity. Together, severe symptoms at time of acute infection were associated with higher IFN-ɣ production, antibody titers, and the risk of hair loss.

## Discussion

This study evaluated the usage of an IGRA as a diagnostic test for CMI against SARS-CoV-2 in a large cohort of 522 individuals within the first months of the pandemic prior to the main vaccine program in Germany. At the time of data acquisition there was still a low SARS-CoV-2 seroprevalence of approximately 4% with 3.362.316 and 71.465 cases reported until April 28^th^ 2021 in Germany and Hamburg, respectively (20). Accordingly, in this time window healthy controls with low likelihood of previous asymptomatic SARS-CoV-2 infection or vaccination could be recruited. This is critical in order to determine cutoff values for CMI tests as with increasing seroprevalence and presence long-term adaptive cellular immunity against SARS-CoV-2 there will be a lack of adequate samples as negative controls for assessment of new diagnostic applications in the future.

Although several studies have reported on SARS-CoV-2 CMI using IGRA- or ELISpot-based test systems (21–23), the data presented here make its contribution since i) a large number of samples were analyzed for CMI and antibodies, ii) there was a long time period between infection to analysis, iii) a comparative use of S or NC peptides was performed, and iv) test results could be correlated to acute and late clinical symptoms. Accordingly, this study provides additional aspects on how to use IGRA-based CMI tests in the future to evaluate adaptive immunity directed to SARS-CoV-2. For example, for distinguishing between individuals inoculated with S-based vaccines or a prior SARS-CoV-2 infection the use of peptides derived from other than the S protein will be useful and NC peptides match these requirements. Although serologic testing with corresponding specificity can similarly provide this information, individuals who cannot acquire a regular humoral immune response could benefit from the availability of a CMI-based diagnostic test. Moreover, individuals with inborn or acquired immune-deficiencies, such as individuals receiving chemotherapy, a B cell depleting medication, or individuals living with human immunodeficiency virus infection may be tested for presence of cellular immunity *via* this IGRA-based assay. Furthermore, in newborns there is a diagnostic window where both, false negative, due to lack of antibody response, or false positive serologic testing, as a result of pre- or postnatally transmitted maternal immunoglobulin, are possible (24). In contrast, early life T cell immunity can be detected in the postnatal phase because fetal T cells exist from the 2^nd^ trimester. Indeed, as a proof of principle, this has been shown for congenital HCMV infection (25), and thus testing for CMI may be a useful diagnostic approach to detect SARS-CoV-2 infection in newborns (26). Nevertheless, T cell immunity and IFN- γ responses may be lower in the early life phase (27) and different cut-off values may apply for the CMI tests.

We found a higher baseline IFN-γ concentration in convalescent individuals than in the healthy control group even months after acute SARS-CoV-2 infection. Interestingly, HCMV^+^ individuals also had a higher IFN-γ plasma concentration than HCMV^-^ study participants. HCMV, a β-herpesvirus is an opportunistic pathogen that leads to lifetime persistent infection (28) whereas SARS-CoV-2 is considered to cause only transient infection. Thus, a chronic inflammatory response with high frequency of activated lymphocytes and according elevated blood baseline IFN-γ concentration in HCMV^+^ individuals is comprehensible. In contrast, residual immune activation in convalescent SARS-CoV-2 patients with elevated blood baseline IFN-γ concentration even months after acute infection is not expected and future studies will need to decipher the causes and consequences of this observation.

CMI NC testing exhibited performance characteristics with almost comparative features as a commercially available anti-NC antibody-based diagnostic test. In contrast to serologic assays which apparently exhibit comparable performance independent from antibody specificity to S or NC (29), the use of S peptides for CMI led to inferior specificity for detection of SARS-CoV-2 infection. Apparently, in a recent study dominant immunogenic HLA-DR SARS-CoV-2 T cell epitopes were mainly found to be derived from the NC protein (30). Thus, we speculate that the observed higher median production of IFN-γ (0.656 versus 0.260 [IU/ml], *p* < *0.0001)* after stimulation with NC or S peptides, respectively was due to more frequent recognition of NC-specific CD4 T cells in peripheral blood. Accordingly, a higher magnitude of IFN-γ responses in ratio to healthy controls led to a better discrimination performance of the CMI NC test. However, we also found several samples to be tested falsely positive only with the CMI S but not with CMI NC or anti-NC. Children are supposed to develop cross-reactive immunity to SARS-CoV-2 after exposure to endemic human coronaviruses (hCoVs) with a deviated and sustaining response to S peptides (31). However, to our knowledge, this has not been shown for adults and we can only speculate that prior infection with hCoVs may have caused these cellular immune responses to S peptides (32, 33). Similarly, individuals who were assigned to the healthy control group but measured as positive by all CMI and anti-NC tests may have experienced a prior asymptomatic SARS-CoV-2 infection. It needs to be mentioned that individuals included in the current study were infected with early variants of SARS-CoV-2 and only a minor fraction may have been exposed to the variant of concern Alpha (B1.1.7), whereas the variants Delta and Omicron were not present in Germany at this time. The emergence of new variants affect the performance of anti-S more than that of anti-NC antibody tests (34), whereas T cell immunity is less affected by these virus mutations and preserved after natural infection and vaccination (11, 35). This argues that CMI-based diagnostic tests may have favorable performance if compared to serologic test for assessment of infection history with different SARS-CoV-2 variants. Nevertheless, at time of data acquisition there was a SARS-CoV-2 seroprevalence below 1% in Germany, there were very few circulating variants, and accordingly the likelihood for silent re-infection in the convalescent group was rather low. Future studies will need to assess the effects of virus re-exposure and vaccination on CMI testing.

We found high WBC counts in peripheral blood to reduce lymphocyte responses to SARS-CoV-2 peptide stimulation leading to several false negative CMI measurements. Although data on differential blood counts was not available in this study, high WBC counts are usually the result of increased numbers of neutrophils and one can speculate that these cells may be causative for the false negative CMI test results. Indeed, although under debate, there is evidence that neutrophils can interfere with cytokine production of T cells (36). However, in the current study we did not assess which and how leukocytes may interfere with the CMI assay. Nevertheless, leukocyte numbers in peripheral blood need to be considered as a confounding factor for borderline test results in future CMI test applications.

There was a positive correlation between anti-NC titers, age, and several conditions found in patients with metabolic syndrome such as high BMI, dyslipidemia, diabetes, and increased cystatin C plasma concentration as an indicator for reduced glomerular filtration rates. Obesity and peak anti-SARS-CoV-2 titers after infection was observed previously (37). In contrast, high age and obesity is associated with lower antibody titers in response to SARS-CoV-2 mRNA vaccines (38). Moreover, antibody titers were observed to not differ between diabetic and healthy individuals in the early phase after SARS-CoV-2 infection (39). Thus, the observed higher anti-NC titers in this study need confirmation by additional study cohorts with focus on long-term immunity and further investigation to understand the underlying mechanism. Interestingly, we found severe symptoms during acute SARS-CoV-2 infection to be associated with loss of hair and individuals with this post COVID-19 symptom exhibited higher anti-NC titers. This observation is in line with recent reports of transient hair loss after severe COVID-19 (40, 41), a known phenomenon observed after severe infectious disease (42). In this cohort, there was no association between CMI and post COVID-19 symptoms. However, a severe clinical course in the acute infection phase led to subsequent higher T cell IFN-γ secretion and future studies need to assess the relevance of this observation for potential long-term organ alterations and the post-acute COVID-syndrome (43).

A gradual decay in SARS-CoV-2-specific antibody titers in convalescent individuals has been described (44) and we could confirm this observation in the present study. We also found lower antibody and IFN-ɣ levels in samples obtained from individuals who were infected more than half a year before blood withdrawal than those with a more recent infection (**Figure 4B**) indicating that both, antibody and T cell responses wane over time. This statistical analysis needs to be interpreted with caution as there were 3.3-fold more individuals in the late than in the early response group. Moreover, there was no significant decay in IFN-ɣ production when including all samples without subdividing into the two groups (**Figure 4A**) and this is in line with observations by recent studies (45). Together, this argues that immunity to SARS-CoV-2 declines over time but T cell immunity likely outlasts plasma antibody titers. This could be a significant advantage of CMI to antibody testing in conditions where long-term adaptive immunity needs to be assessed.

Together, we characterized an in-house developed laboratory test which exhibits excellent test performance, is suitable for high through-put routine diagnostics, and applicable to laboratories with equal instrument equipment. The use of this diagnostic test should be considered in clinical conditions with impaired antibody responses such as innate and acquired immune deficiencies or the early life phase where endogenous cannot be differentiated from maternal immunoglobulin. Moreover, detection of CMI may be useful for future pandemic-related questions such as assessment of long-term adaptive immunity and clinical course after SARS-CoV-2 re-infection.

## Data availability statement

The datasets presented in this article are not readily available because requests for HCHS dataset need to be addressed to the corresponding committee. Requests to access the datasets should be directed to RT, r.twerenbold@uke.de.

## Ethics statement

The studies involving human participants were reviewed and approved by Hamburg Chamber of Medical Practitioners (PV5131). The patients/participants provided their written informed consent to participate in this study.

## Author contributions

LFB and FRS established the CMI test; LFB, ST, JK, KC, AS, MA, and ML conducted CMI, PCR, and antibody data acquisition; FRS and LFB analyzed the data; IS and RT coordinated the study cohort; EP coordinated study population data; FRS conceived the project and wrote the manuscript. All authors reviewed the manuscript. All authors contributed to the article and approved the submitted version.
